# Strengthening the Direct Care Workforce: Policy and Practice Solutions for Sustainable Aging Services

**DOI:** 10.1093/geroni/igaf122.619

**Published:** 2025-12-31

**Authors:** Julie Lowenthal

**Affiliations:** Ageways Nonprofit Senior Services, Berkley, Michigan, United States

## Abstract

The direct care workforce (DCW) is essential to aging services, yet persistent workforce shortages, high turnover (40-60% annually), and low wages threaten its stability. Economic insecurity, limited career advancement, inadequate training, and transportation barriers—key social determinants of health (SDOH)—exacerbate these challenges, impacting worker retention and care quality. This paper explores workforce trends, the intersection of SDOH, and evidence-based strategies to improve workforce sustainability. Key solutions include increasing Medicaid reimbursement rates to support wage growth, expanding training and credentialing opportunities, and leveraging public-private partnerships to enhance recruitment and retention. State-level case studies demonstrate that wage increases, career ladders, and technology-driven workforce innovations lead to improved job satisfaction and retention. As the U.S. population aged 65+ surpasses 70 million by 2030, a stable direct care workforce is critical to meeting long-term care demands. This research provides policy recommendations for strengthening workforce recruitment, retention, and career development to ensure high-quality care for older adults and individuals with disabilities.

